# Chronic Restraint Stress-Induced Muscle Atrophy Leads to Fatigue in Mice by Inhibiting the AMPK Signaling Pathway

**DOI:** 10.3390/biomedicines9101321

**Published:** 2021-09-26

**Authors:** Zhi Wang, Tianji Xia, Suwei Jin, Xinmin Liu, Ruile Pan, Mingzhu Yan, Qi Chang

**Affiliations:** Institute of Medicinal Plant Development, Chinese Academy of Medical Sciences and Peking Union Medical College, Beijing 100193, China; wangzhimax@163.com (Z.W.); m13521515301@163.com (T.X.); jinsuwei0202@163.com (S.J.); liuxinmin@hotmail.com (X.L.); rlpan@implad.ac.cn (R.P.)

**Keywords:** chronic restraint stress, fatigue, muscle atrophy, mitochondrial dysfunction, mitophagy, AMPK

## Abstract

Currently, an increasing number of people are suffering from fatigue due to the state of their lifestyles, such as sedentary work in a relatively small space, irregular sleep patterns, or the lack of movement and exercise. The present study was designed to simulate the occurrence of fatigue in the above populations through a chronic restraint stress (CRS) model, and to reveal its dynamic processes and potential underlying molecular mechanisms. ICR mice were subjected to 8 h of restraint stress each day for 5, 10, or 15 days. It was found that the weight-loaded swimming performance, grip strength, and locomotor activity of the mice all decreased under CRS treatment, and that up to 15 days of CRS induced notable fatigue. Gastrocnemius muscle atrophy and some abnormal biochemical parameters related to fatigue under CRS were observed. Furthermore, transcriptome data showed that the changes in muscle cell metabolism and mitochondrial dysfunction were associated with the AMPK signaling pathway in CRS-treated mice. Western blotting analysis of the AMPK/PGC-1α signaling pathway revealed that CRS could decrease mitochondrial biogenesis and reduce the numbers of type I skeletal muscle fibers in the gastrocnemius of mice. CRS could also block the protective mitophagic flux to inhibit the abnormal clearance of damaged mitochondria. Our study suggests a critical link between muscle atrophy and CRS-induced fatigue in mice, suggesting that the pharmacological promotion of muscle and mitochondrial function can be used as a treatment for stress-induced fatigue.

## 1. Introduction

Fatigue is a subjective experience of persistent tiredness, weakness, or exhaustion that can be mental, physical, or both [1]. Fatigue can be caused by a number of factors, including but not limited to stress, overwork, overexercise, undersleep, and a variety of diseases, such as cancer, Parkinson’s disease, and many neuroimmunological diseases [2,3,4,5]. With increasing social stress, the prevalence of fatigue continues to rise, to the point that fatigue has become a widespread societal health problem [6]. Some epidemiological studies showed that over 50% of respondents to 400 surveys of adults in Detroit claimed fatigue, and the most frequent symptom among Norwegian adults was fatigue (59.7%) [7,8]. Moreover, a survey in England and Wales suggested a substantial increase in mortality from suicide in people with fatigue [9]. Importantly, fatigue reduces quality of life, decreases the efficiency of work, and increases the risk of accidents, resulting in a huge social burden [10,11].

Mitochondria are vital sites of respiration and oxidative phosphorylation, and are known as cellular “power plants”. Mitochondria play a very important role in energy metabolism; moreover, fatigue was found to be a very common phenomenon in patients with mitochondrial disease [12,13]. Healthy mitochondria are particularly important to skeletal muscle fitness [14]. Skeletal muscles need to consume much energy when they are exercised, and fatigue will occur when energy supply processes are dysregulated. AMP-activated protein kinase (AMPK) is a major sensor of energy demand in exercise and muscle contraction. Activation of the AMPK signaling pathway increases catabolism and regulates energy homeostasis. Mitochondrial biogenesis in skeletal muscle is also regulated by energy demands. According to previous studies, peroxisome proliferator-activated receptor γ coactivator 1α (PGC-1α) is activated by AMPK and adjusts mitochondrial biogenesis and normal functioning as a master regulator [15]. The elimination of damaged mitochondria is also important, and mitophagy can help maintain fresh mitochondrial function. These biological processes are primarily regulated by AMPK.

Chronic restraint stress (CRS) is a method used to induce physiological responses in an animal by restricting its free movement in a narrow space. Many studies have demonstrated that CRS can cause various dysfunctions and damage in animals, including anxiety, depression, and memory impairment [16,17,18]. A few reports suggest that CRS may induce fatigue [19,20]. CRS can simulate the general fatigue of modern society, which is caused by sedentary work in a small environment, an irregular schedule, and a lack of activity. Moreover, interestingly, some research has shown that acute and chronic stressors influence normal mitochondrial function [21]. Therefore, it is reasonable to presume that CRS may reduce exercise endurance by causing mitochondrial dysfunction in the gastrocnemius.

However, there are still many issues that must be addressed regarding the effect of CRS in inducing fatigue, including (1) the specific dynamic process of CRS-induced fatigue in mice, (2) whether CRS-induced fatigue in mice is related to gastrocnemius muscular and mitochondrial dysfunction, and (3) the associated molecular mechanisms.

## 2. Materials and Methods

### 2.1. Animals and CRS Treatments

Male ICR mice (4 weeks, weight 23 ± 1 g) were purchased from Vital River Laboratories (Beijing, China). The mice were housed 6 per cage under a constant temperature (22–25 °C) and humidity (50–60%) with a 12 h/12 h light/dark cycle. Control mice were forbidden access to food and water when the stress groups were under restraint. The experimental procedures were approved by the Animal Ethics Committee of the Institute of Medicinal Plant Development, Chinese Academy of Medical Sciences, and Peking Union Medical College.

**Figure 1:**
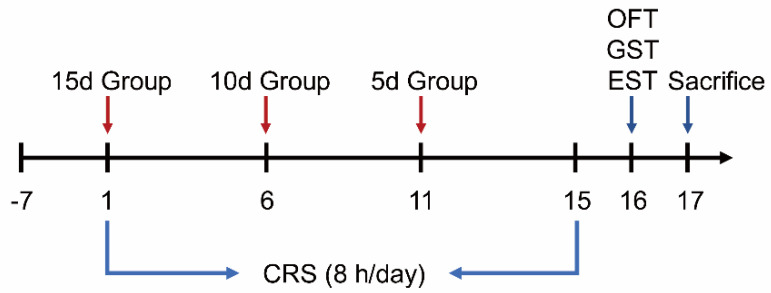
Experimental design of CRS-induced fatigue. Group CRS (15 day) was restrained on day 1, while group CRS (10 day) and group CRS (5 day) were restrained on day 6 and 11, respectively.

After adapting to the environment for 7 days, the mice were divided into 4 groups according to body weight and grip strength, with one control group and three CRS groups, and 12 mice in each group. The experimental design is shown in Figure 1. The mice in the CRS groups were restrained in ventilated tubes (diameter 30 mm; length 120 mm) for 8 h/day (10:00–18:00) for 5, 10, or 15 days.

### 2.2. Behavioral Tests

Exhaustive swimming test (EST): Mice were loaded with 8% body weight of lead attached to the root of the tail, and then individually placed in a swimming box (diameter 20 cm) containing water at a temperature of 25 ± 1 °C and with a depth over 25 cm. The swimming behavior of the mice was recorded from the beginning of swimming to exhaustion, which meant that the mice were unable to return to the surface of the water due to fatigue after 7 s. The first successive sinking time was the first time when the head of the mouse was completely submerged for more than two successive times in the swimming process.

Grip strength test (GST): Mice were placed on a grip strength meter from Yiyan Technology (YLS-13A, Jinan, China). When the mice grasped the grid, they were slowly pulled backwards, with the pull gradually increased until the mice loosened their claws. The high-precision force sensor of the tester recorded the maximum grip. The grip strength of each mouse was measured three times to take an average.

Open field test (OFT): Mice were placed in a locomotor activity tester (30 cm × 28 × 35 cm) to analyze their locomotor activity. Animal movement distance and time in the bare arena within 5 min were recorded after adapting to the environment for 2 min.

### 2.3. Biological Sample Collection

The day after the behavioral tests, the mice were forced to swim (25 ± 1 °C, 25 cm depth) for 30 min. Subsequently, the mice were dried with a clean towel and allowed to rest for 30 min in their cages. After pentobarbital sodium anesthesia, blood was collected from the abdominal aorta and centrifuged to obtain serum (3000 rpm, 15 min, 4 °C). Mice were euthanized by cervical dislocation, and then the liver and hindlimb gastrocnemius were removed and rapidly placed in liquid nitrogen. All of the samples were stored at −80 °C until analysis.

### 2.4. Determination of Biochemical Parameters

The levels of glucose (Glu), urea, lactate dehydrogenase (LDH), and aspartate aminotransferase (AST) in the serum were detected using commercial kits (BioSino, Beijing, China). Serum lactic acid (LA), liver glycogen, and muscle glycogen were tested using commercial kits (Jiancheng, Nanjing, China).

### 2.5. Transcriptomic Analysis

Total RNA was extracted from control and CRS (15 days) mice using TRIzol (Solarbio, Beijing, China) and subjected to RNA-Seq analysis. The quality of RNA was evaluated using a NanoDrop and an Agilent 2100, and then RNA was sequenced using an Illumina sequencer. The fragments per kilobase of exon model per million mapped reads (FPKM) of gene expression in each sample were calculated by HTSeq. Then, *p*-values were obtained from a negative binomial distribution test and adjusted using FDR (BH). Differentially expressed genes (DEGs) were screened by adjusted *p*-values of less than 0.05.

### 2.6. Western Blotting Analysis

Gastrocnemius tissues were homogenized and lysed in cold RIPA buffer (Solarbio, Beijing, China) containing 1% phosphatase and protease inhibitor cocktail (CWBIO, Beijing, China). Protein concentrations in the homogenates were quantified using a BCA protein assay kit (Solarbio, Beijing, China) and adjusted to the same concentrations. Twenty-five to fifty micrograms of denatured proteins and three microliters of multicolor protein marker (CWBIO, Beijing, China) were subjected to SDS-PAGE and transferred to a nitrocellulose membrane (0.2 μm). Membranes were blocked in 5% nonfat milk for 1.5 h and incubated overnight with specific primary antibodies at 4 °C. Subsequently, the membranes were incubated with the appropriate horseradish peroxidase-conjugated secondary antibodies (CWBIO, Beijing, China) for 1.5 h at room temperature (goat anti-rabbit and goat anti-mouse IgG H&L, at 1:5000 dilution). Protein expression was visualized using the enhanced chemiluminescence method in a gel imaging system (Bio-Rad, Hercules, CA, USA). The signal intensities were normalized to the corresponding total protein or GAPDH.

The antibodies used were as follows: GAPDH (AC001), MYH7 (A7564), and PINK1 (A7131) were purchased from ABclonal (Wuhan, Hubei, China); AKT(#4691), Phospho-AKT (Ser473) (#4060), AMPK (#5831), Phospho-AMPKα (Thr172) (#2535), mTOR (#2983), Phospho-mTOR (Ser2448) (#5536), Phospho-ULK1 (Ser757) (#14202), and LC3A/B (#12741) were purchased from Cell Signaling Technology (Denver, MA, USA); CYT-C (ab133504), TOMM20 (ab186735), OXPHOS (ab110413), PGC1-α (ab54481), and TFAM (ab252432) were purchased from ABCAM (Cambridge, UK); and p62/SQSTM (18420-1-AP) was purchased from ProteinTech (Wuhan, Hubei, China). All antibodies were diluted at a ratio of 1:1000.

### 2.7. Histological and Immunohistochemistry Analyses of Muscle

Gastrocnemius muscles were fixed in 4% paraformaldehyde-buffered solution for 24 h and then embedded in paraffin. Five-micron-thick sections of the gastrocnemius were cut and stained with hematoxylin and eosin (H&E). The diameter and cross-sectional area of the muscle fibers were quantified by 2 representative 10× sections from each of the 3 samples. For MYH7 immunohistochemistry, gastrocnemius muscle sections were stained with a rabbit polyclonal anti-MYH7 antibody at a 1:50 dilution ratio.

### 2.8. Transmission Electron Microscopy

The gastrocnemius tissues of mice were cut into 2 × 2 mm^2^ slices and quickly fixed in 2.5% glutaraldehyde for 2 h. After being further fixed in 1% osmium tetroxide and 1% tannic acid, the samples were embedded in epoxy resin and cut into 70 nm ultrathin sections. The sections were double-stained with uranyl acetate and lead citrate and then observed with an electron microscope (Hitachi HT7700, Tokyo, Japan) at 80.0 kV.

### 2.9. Statistical Analysis

Quantification of blots was performed using ImageJ software (Version 1.8.0, National Institutes of Health, Bethesda, MD, USA), and the results were normalized to GAPDH. To analyze muscle atrophy, the cross-sectional diameter and area of the muscle fiber were estimated by ImageJ software. Differences between two groups were analyzed using Student’s t-test. One-way ANOVA was used for comparisons of more than two groups. When data deviated from a normal distribution, nonparametric tests were used. Calculations were performed using SPSS 25.0 statistical software (IBM Corp., Armonk, NY, USA). All data are presented as the mean ± standard error of the mean (SEM), with significance set at *p* < 0.05. Graphs were drawn using GraphPad Prism (version 7.0, San Diego, CA, USA).

## 3. Results

### 3.1. CRS Induced Fatigue and Its Specific Dynamic Process in Mice

**Figure 2:**
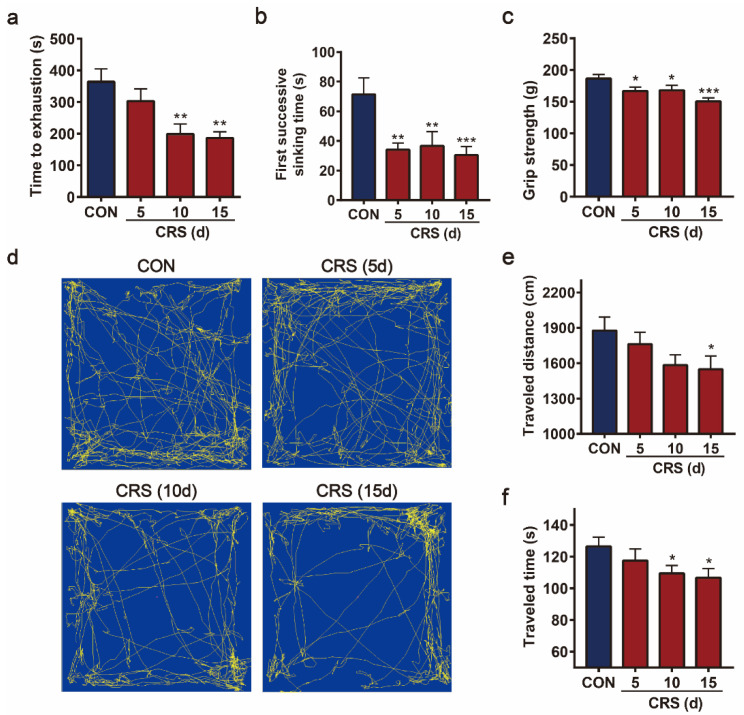
The effects of CRS on the behaviors of mice in the exhaustive swimming test (EST), grip strength test (GST), and open field test (OFT). Exhaustion time (**a**) and first successive sinking time (**b**) in EST. Grip strength in GST (**c**). Motion trail (**d**), traveled distance (**e**), and traveled time (**f**) in the OFT. Data are presented as the mean ± SEM (n = 10–12), * *p* < 0.05, ** *p* < 0.01, *** *p* < 0.001.

To examine whether CRS causes fatigue in mice, and to further explore the corresponding dynamic changes during CRS, the locomotor activity, muscle strength, and swimming endurance of mice were monitored under different stress durations. In the EST, the time to exhaustion decreased after CRS treatment. Specifically, 10 and 15 days of CRS significantly reduced the swimming time, to 54.5% and 51.2% of the vehicle-treated control group, respectively (Figure 2a). Similarly, the first successive sinking time was significantly reduced from 5 days of CRS to 47.7%, 51.3%, and 42.6% of control mice after CRS treatment for 5, 10, and 15 days, respectively (Figure 2b). The grip strength of CRS mice was also significantly decreased after 10 and 15 days of CRS treatment (Figure 2c). In the OFT, the total traveled distance and traveled time decreased with increasing duration of CRS (Figure 2d). Compared with the control group, 5- and 10-day CRS-treated mice showed downward trends in traveled distance. These trends became significant at 15 days after CRS treatment (Figure 2e). Consistent with the traveled distance, traveled time was significantly reduced at 10 and 15 days in CRS-treated mice (Figure 2f). Together, these data demonstrate that 10 days of CRS could cause fatigue in mice, and the extent increased with the duration to 15 days, at which time the fatigue state was stable in mice.

### 3.2. CRS Induced Muscle Atrophy and Disorder in Gastrocnemius Structure and Function

**Figure 3:**
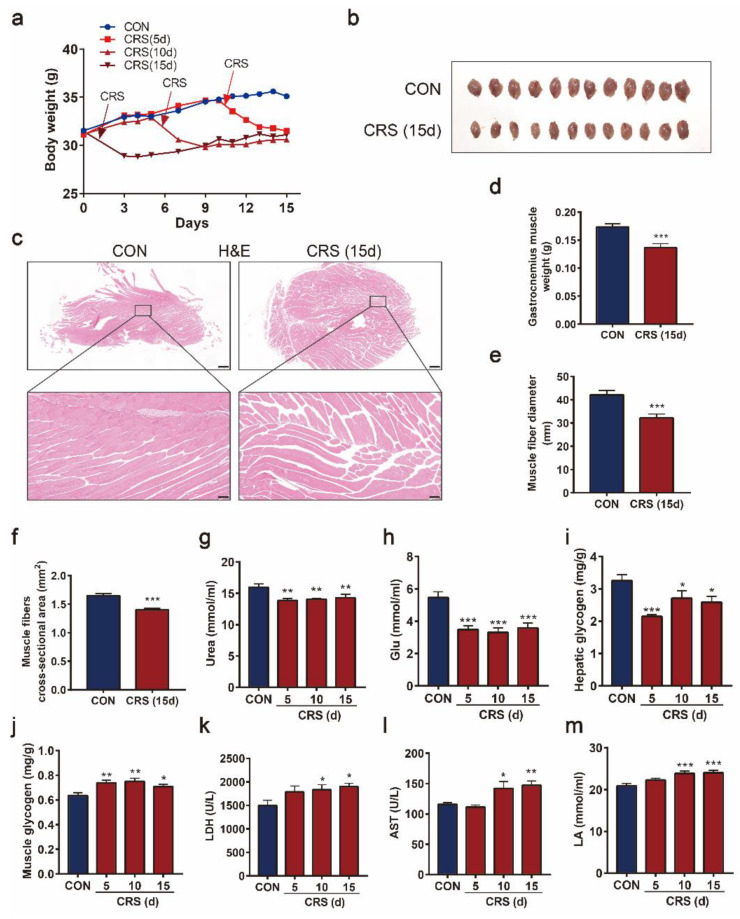
Effects of CRS on muscle function and fiber type. Effects of CRS on body weight (**a**) and anatomical observation of gastrocnemius muscles (**b**). Representative H&E-stained cross-sections (**c**) of gastrocnemius muscles (upper panel, scale bar = 500 μm; lower panel, scale bar = 100 μm). The weight of gastrocnemius muscles (**d**) (n = 12). The muscle fiber diameter (**e**), and the cross-sectional area (**f**) of gastrocnemius muscles, n = 3 per group. The serum levels of urea (**g**), Glu (**h**), LDH (**k**), AST (**l**), and LA (**m**). Hepatic (**i**) and muscle (**j**) glycogen in mice (n = 10–12). Data are presented as the mean ± SEM, * *p* < 0.05, ** *p* < 0.01, *** *p* < 0.001.

To understand the mechanism underlying the fatigue induced by CRS, the muscle—which is directly related to exercise endurance—was investigated. Body weights (Figure 3a) and serum urea levels (Figure 3g) in CRS mice were significantly reduced, implying that muscle loss and protein malnutrition might occur. This was confirmed by the decreased gastrocnemius muscle weight after 15 days of CRS compared with the vehicle control (Figure 3b,d). The muscle fibers of the gastrocnemius in control mice were neatly arranged and clearly organized, whereas those in CRS mice were severely divided, polygonally atrophic, and irregularly shaped (Figure 3c). Quantitative analysis revealed that the diameter and cross-sectional area of the muscle fibers had markedly decreased after 15 days of CRS (Figure 3e,f). The levels of Glu (Figure 3h) and hepatic glycogen (Figure 3i) in CRS mice were decreased in comparison with those of control mice. However, muscle glycogen storage (Figure 3j) in CRS-treated mice was significantly higher than that in control mice, suggesting that muscle glycogen utilization was impaired. Further investigation found that these changes might be due to muscle cell damage, as demonstrated by increased serum levels of LDH (Figure 3k) and AST (Figure 3l). Moreover, the serum levels of LA (Figure 3m) significantly increased in mice after 10 or 15 days of CRS treatment, indicating lower aerobic capacity and increased glycolytic flux in muscle cells. Taken together, these data demonstrate that CRS could reduce muscle mass and impair muscle structure and function in mice.

### 3.3. CRS Induced Changes in the Gene Expression Profile in the Gastrocnemius of Mice

**Figure 4:**
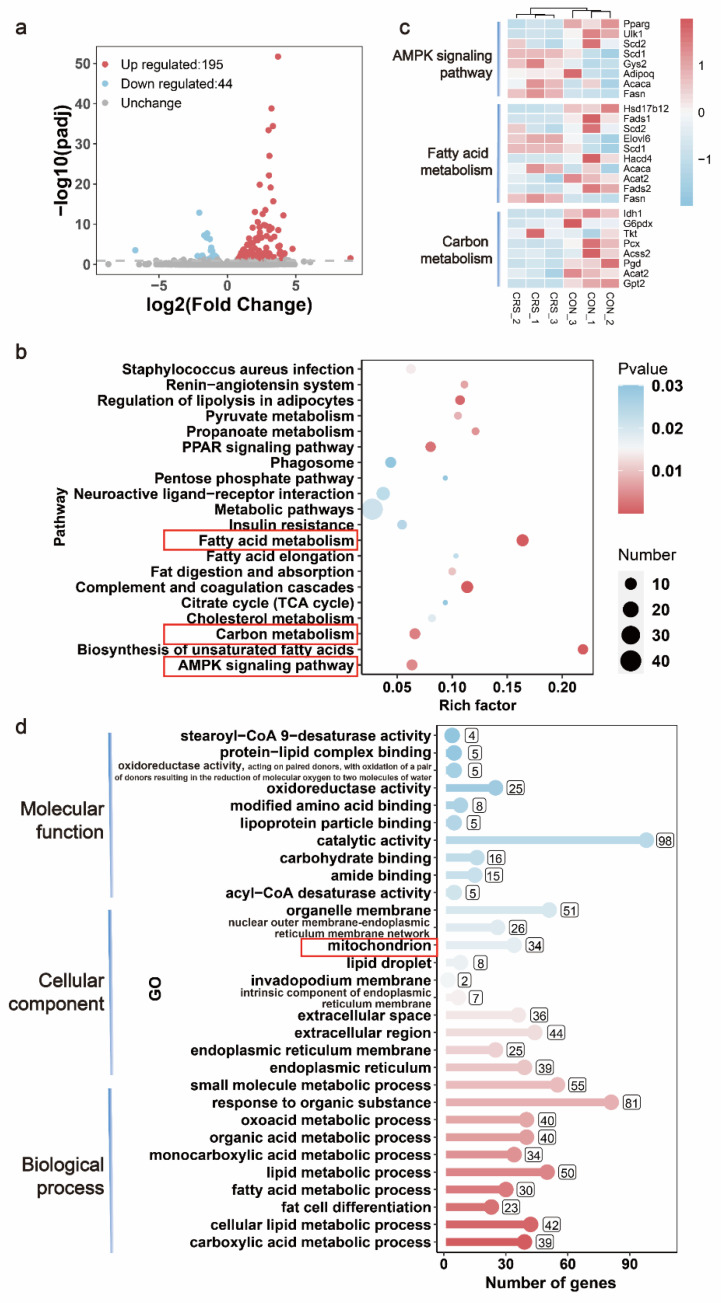
Effects of CRS on gene expression in the gastrocnemius of mice, by RNA-Seq data. Volcano map of the DEGs and their regulation (**a**). Heatmap of the AMPK signaling pathway, fatty acid metabolism, and carbon-metabolism-related DEG expression (**b**). KEGG (**c**) and GO (**d**) analyses of 239 specific DEGs in the gastrocnemius. n = 3 per group.

To systematically examine the effects of CRS on murine muscle to drive fatigue, the gastrocnemius muscles of control and 15-day CRS-treated mice were analyzed via transcriptome sequencing. A total of 239 specifically differentially expressed genes (DEGs) were found in the control and CRS-treated groups. Among them, 195 genes were significantly upregulated and 44 genes were significantly downregulated by CRS (Figure 4a). Kyoto Encyclopedia of Genes and Genomes (KEGG) pathway enrichment results revealed that the AMPK signaling pathway was significantly regulated. Carbon-metabolism-related pathways—such as the pentose phosphate pathway (PPP), citrate cycle (TCA cycle), and pyruvate metabolism—were enriched (Figure 4c). The results showed that glucose-6-phosphate flows to the PPP, resulting in compensatory upregulation of fatty acid metabolism for energy supply. A heatmap based on the KEGG results revealed that genes related to the AMPK signaling pathway, fatty acid metabolism, and carbon metabolism were significantly regulated by CRS treatment (Figure 4b). Molecular function of Gene Ontology (GO) analysis showed that the activities of oxidoreductase and other molecules were enriched. Cellular components revealed that a large number of these DEGs encoded mitochondrial proteins, and many lipid metabolic-process-related metabolic pathways were enriched in biological processes (Figure 4d).

### 3.4. CRS Induced Mitochondrial Loss and Dysfunction in Muscle Cells

**Figure 5:**
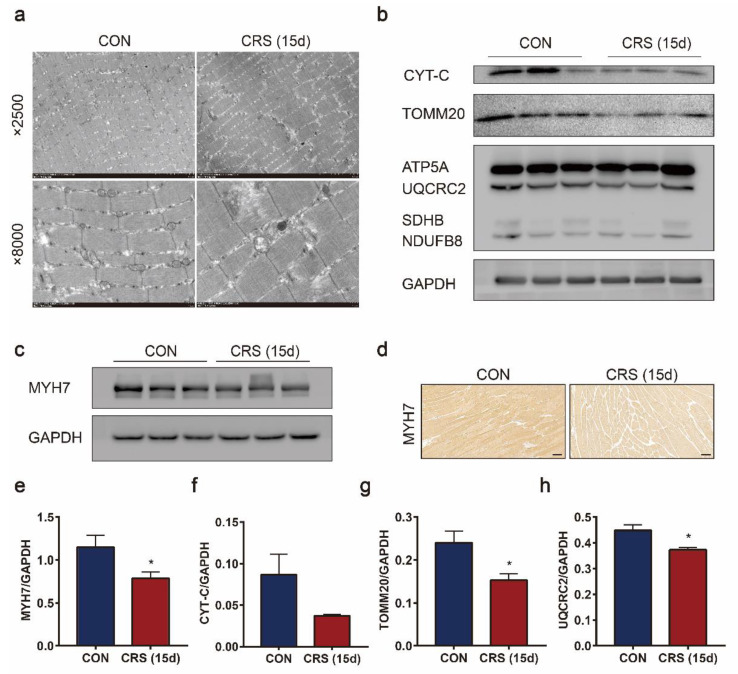
Effects of CRS on mitochondrial dysfunction in the muscle of mice. Electron micrographs of the CON and CRS gastrocnemius muscles (**a**). Immunoblotting of CTT-C, TOMM20, OXPHOS (**b**) and MYH7 (**c**) in the gastrocnemius muscle. Representative images of immunohistochemical staining for MYH7 (**d**) of the CON and CRS gastrocnemius muscles (scale bar = 100 μm). And the quantification of MYH7 (**e**), CTT-C (**f**), TOMM20 (**g**), and UQCRC2 (**h**) protein expression. Data are presented as the mean ± SEM (n = 3), * *p* < 0.05.

Mitochondria play critical roles in regulating muscle cell energy supply. We speculate that mitochondria are involved in CRS-induced muscle dysfunction based on the results from transcriptome sequencing analysis. To explore this hypothesis, the ultrastructure of the gastrocnemius was observed via electron microscopy (EM). Mitochondria in the gastrocnemius of control mice displayed a standard size and were uniformly distributed (Figure 5a). In contrast, mitochondria in CRS-treated mice were fragmented and maldistributed, and their total number was decreased. Indeed, type I skeletal muscle fibers, which are rich in mitochondria, were reduced by CRS treatment, as revealed by decreased MYH7 in the gastrocnemius (Figure 5c–e). Consistently, the decreased expression of the mitochondrial marker proteins CYT-C and TOMM20 suggested that the number of mitochondria was substantially reduced by CRS (Figure 5b). Moreover, the significant decrease in the UQCRC2 levels (Figure 5b) implied the suppression of oxidative phosphorylation.

### 3.5. CRS Induced Muscle Dysfunction and Mitochondrial Biogenesis Decrease via AMPK/PGC-1α

**Figure 6:**
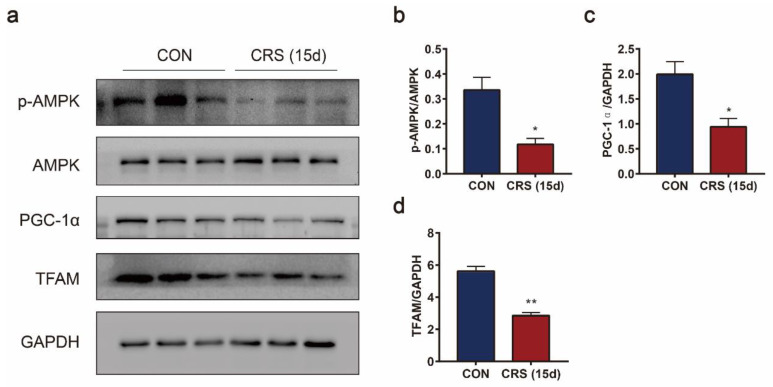
Effects of CRS on the AMPK/PGC-1α signaling pathway lead to muscle and mitochondrial dysfunction. Immunoblotting of p-AMPK, AMPK, PGC-1α, and TFAM (**a**) in the gastrocnemius muscle, and the quantification of p-AMPK/AMPK (**b**), PGC-1α (**c**), and TFAM (**d**) protein abundance. Data are presented as the mean ± SEM (n = 3), * *p* < 0.05, ** *p* < 0.01.

AMPK is an important regulatory protein involved in mitochondrial quality control by regulating mitochondrial biogenesis, fusion/fission, and energy generation. To elucidate whether CRS-induced muscle and mitochondrial dysfunction were related to AMPK, the expression of p-AMPK was determined, and was found to be significantly reduced by CRS (Figure 6a). As a consequence, the expression of PGC-1α and its downstream factor TFAM also decreased (Figure 6a). Thus, our data suggest that CRS-induced mitochondrial dysfunction in the gastrocnemius is associated with the AMPK/PGC-1α signaling pathway.

### 3.6. CRS Blocked Mitophagy in Muscle Cells

**Figure 7:**
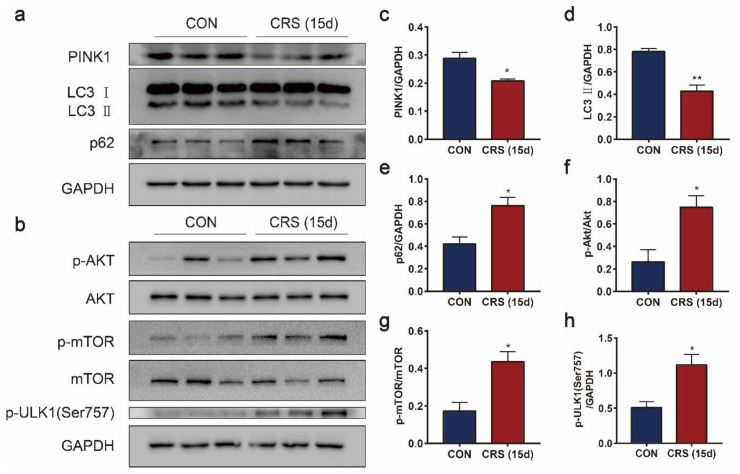
Effects of CRS on mitophagy in the gastrocnemius muscle. Immunoblotting of PINK1, LC3, and p62 in the gastrocnemius muscle (**a**). Immunoblotting of p-AKT, AKT, p-mTOR, mTOR, and p-ULK1 (Ser757) in the gastrocnemius muscle (**b**). Quantification of PINK1 (**c**), LC3-II (**d**), p62 (**e**), p-AKT/AKT (**f**), p-mTOR/mTOR (**g**), and p-ULK1 (Ser757) (**h**) protein abundance. Data are presented as the mean ± SEM (n = 3), * *p* < 0.05, ** *p* < 0.01.

Mitophagy is an important mitochondrial quality control mechanism that eliminates damaged mitochondria. Compared with the control group, the mitophagy was inactivated in the gastrocnemius muscle after CRS treatment, as revealed by the decreased expression of PINK1 (Figure 7c), LC3 (Figure 7d) and the elevated expression of p62 (Figure 7e), resulting in the accumulation of damaged mitochondria (Figure 7a). Further investigation of the AMPK/mTOR pathway revealed the causes of blocked mitophagy. The expression of p-AKT (Figure 7f) and p-mTOR (Figure 7g) increased markedly in CRS-induced mice (Figure 7b), and upregulated p-ULK1 (Ser757) (Figure 7h), resulting in the destruction of the interaction between ULK1 and AMPK. Taken together, the inhibition of AMPK activation could not promote mitophagy by inhibiting the mTOR pathway.

## 4. Discussion

As a modeling method, CRS forces animals to undergo continuous and predictable stress to mimic everyday human stress, such as daily repetition of a stressful job and social and familial stresses. CRS has been shown to cause behavioral, gene expression, protein, and brain function changes similar to those in patients with cognitive impairment and depression. However, there is limited evidence for CRS causing fatigue and decreasing exercise performance. To date, only two studies performed by Park et al. have applied CRS to induce fatigue in mic [19,20]; they revealed the occurrence of CRS-induced fatigue from the perspective of neuroinflammation and brain hormones. However, whether CRS induces peripheral muscle dysfunction in mice and the related molecular mechanisms remains unclear. The dynamic process of fatigue induced by CRS, and its mechanism in muscle and mitochondrial dysfunction, are also still unclear. It is necessary to study how chronic stress that may occur in daily life leads to fatigue. Therefore, in this study, we analyzed the effects of CRS on fatigue and its dynamic process in mice suffering CRS for 8 h/day for 5, 10, and 15 days. In the EST, it was found that the development of fatigue was positively correlated with the time of CRS exposure. Mice showed insufficient endurance during exercise after treatment with 10 and 15 days of CRS. Consistently, CRS-induced mice showed a decrease in muscle strength in the GST. Stress and physical activity are believed to be interrelated; stress may cause muscle fatigue and reduced muscle contraction ability, while physical exercise can counter stress [22]. However, in clinical observation, fatigue may reduce people’s willingness to exercise actively in a quiet state [23]. This was also observed in our OFT results; locomotor activity significantly decreased after treatment with CRS for 15 days. This suggests that we need to overcome the unwillingness to do physical activity when we are under stress, and maintaining different types of physical activity—such as high-intensity interval training (HIIT), resistance, and endurance—may help in resisting fatigue. To conclude, a stable fatigue model of mice was constructed at 15 days of CRS.

Skeletal muscle atrophy is the consequence of protein overdegradation and muscle tissue loss [24]. In our study, we found that the level of urea was decreased and gastrocnemius muscles were irregularly atrophied in mice after CRS treatment. A recent study demonstrated that chronic stress induced the lowering of fasting glucose plasma concentration, as well as the imbalance of glucose homeostasis [25]. When sports are played, there is a constant need for energy; thus, the players’ sports endurance is closely related to the storage of energy materials [26]. In the present study, it was found that serum glucose and liver glycogen storage of the mice in the CRS group were notably lower than those in the control group after exercise. The level of muscle glycogen in CRS mice was higher than that in control mice, indicating that its utilization was blocked [27]. In addition, serum lactic acid accumulation was greater in CRS mice than in control mice, which might be related to the increased muscle anaerobic oxidation in mice caused by mitochondrial dysfunction. After strenuous exercise, the oxidative metabolism of the body is enhanced, and a large number of free radicals are produced in skeletal muscle, the heart, and other tissues, which can destroy cell membranes and various organelles, thus damaging the normal metabolic function of cells and causing fatigue [28]. Similarly, LDH and AST levels in serum were significantly increased after exercise, revealing that mice with CRS-induced fatigue will suffer greater muscle and myocardial cell damage during exercise.

How might CRS contribute to muscle atrophy and fatigue? Transcriptomic analysis reminds us that mitochondria may be damaged so that glucose flows to the pentose phosphate pathway (PPP), which is consistent with Fan’s previous findings [29]. Transcriptome analysis also indicated that the AMPK signaling pathway may be a key pathway in the development of CRS-induced fatigue. AMPK can play an energy-regulatory function by downregulating anabolic pathways and upregulating catabolic pathways, such as increasing mitochondrial homeostasis, glucose uptake, protein regulation, and fatty acid synthesis [30]. PGC-1α is an important protein downstream of the AMPK pathway that affects glucose metabolism as well as gluconeogenesis from mitochondrial biogenesis and muscle fiber-type switching in muscles [31]. Other studies have shown that activating AMPK/PGC-1α can make muscles more resistant to fatigue and reduce muscular dystrophy damage [32]. In our study, the low phosphorylation of AMPK at Thr172 and decreased expression of PGC-1α found in CRS-induced fatigue mice indicated that the activation of the AMPK pathway was decreased. Some evidence has revealed that medicines can attenuate skeletal muscle atrophy and dysfunction through AMPK/PGC-1α [33,34].

Additionally, when PGC-1α was upregulated, mitochondrial biogenesis increased as a result of the increased expression of TFAM [35]. CRS treatment reduced the expression of mitochondria-specific CYT-C and TOMM20, as well as the mitochondrial biogenesis marker TFAM. These results demonstrate that CRS results in the number of mitochondria decreasing, which reduces the level of oxidative phosphorylation.

PGC-1α also plays an important role in promoting the expression of myoglobin and troponin, and increasing the proportion of type I muscle fibers. Therefore, activation of muscle PGC-1α leads to much more resistance in response to continuous electrical stimulation fatigue [36]. It has been reported that type II muscle fibers contain a higher level of glycogen and produce more lactic acid than type I muscle fibers after exercise [37,38]. Here, we observed that the muscles appeared lighter red, and that the expression of MYH7 was decreased in the gastrocnemius, revealing that the content of type I muscle fibers was reduced in the gastrocnemius because of the lack of PGC-1α under CRS. These findings may explain why CRS-induced mice exhibit lactic acid accumulation and energy material depletion, and subsequently show a lack of endurance.

Moreover, impaired autophagy may contribute to sarcopenia and a reduction in absolute force [39]. We further investigated the clearance process of damaged mitochondria. When mitochondria are damaged, PINK1 regulates the initiation of protective mitophagy and preserving energy metabolism [40]. p62 accumulates in damaged mitochondria, and binds to the LC3B-II protein to form a complex, which directs the autophagosome to the mitochondria, which eventually degrade in lysosomes [41]. The expression of PINK1 and LC3-II decreased, and p62 accumulated in the gastrocnemius of CRS mice, suggesting that mitophagic clearance was blocked. It has been reported that abnormal accumulation of p62 occurs when AMPK activation is absent [42].

According to previous reports, ULK1 promotes mitophagy, and can be activated by AMPK [43]. Thus, AMPK can eliminate defective mitochondria by regulating ULK1, and promote the generation of new mitochondria through PGC-1α, thus maintaining the normal functioning of the mitochondria in organisms [44]. When AKT is activated, it antagonizes the effect of AMPK, activates downstream mTOR, promotes the phosphorylation of ULK1 at the 757 inhibitory site, and inhibits ULK1 activation to inhibit mitophagy [45]. In the absence of intracellular energy materials, AMPK activates and inhibits mTOR activity, reduces the phosphorylation of ULK1 (Ser757), and re-establishes the relationship between AMPK and ULK1 [46]. However, in our study, we found that activation of AMPK was abnormally inhibited, AKT/mTOR was activated, and ULK1 was inactivated in the gastrocnemius of CRS mice. These findings suggest that although mitophagy was initiated in CRS mice, the increased expression of AKT/mTOR/ULK1 (Ser757) resulted in a certain degree of decreased mitophagy that should have been activated. It has been reported that autophagy is suppressed even under autophagy-inducible starvation conditions to remodel muscle by activating mTOR [47]. Other studies have also shown that muscle atrophy can be prevented and treated by upregulating the AKT/mTOR signaling pathway [48]. In contrast, other findings revealed that the mTOR inhibitor rapamycin surprisingly ameliorates muscle function in a number of muscular dystrophies, and overactivation of mTOR leads to sarcopenia [49]. These results suggest that CRS-induced imbalance of mTOR activation in skeletal muscle cannot protect muscle, even leading to muscle loss.

Herein, we proved that the mitochondria of CRS mice were damaged, but that mitophagy was blocked, resulting in mitochondrial dysfunction in the gastrocnemius. CRS-induced fatigue is caused by the inability to carry out normal-intensity energy metabolism, and this is primarily related to inhibition of the AMPK signaling pathway. Moreover, considering that CRS-induced mice showed insufficient endurance, other physical activities that also require the activation of the AMPK signaling pathway to ensure energy supply—such as HIIT and resistance exercise—may also be weakened.

## 5. Conclusions

**Figure 8:**
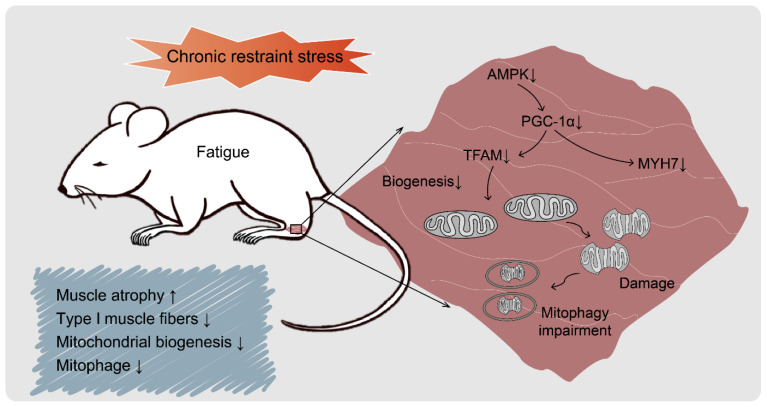
Schematic diagram of the molecular target pathways involved in CRS-induced fatigue. CRS suppressed AMPK phosphorylation and then inhibited AMPK/PGC-1α signaling pathway cascade, thereby subsequently inducing muscle atrophy, decreasing type I muscle fibers, decreasing mitochondrial biogenesis, and suppressing protective mitophagy of the gastrocnemius.

Overall, the present study provides insights into the specific dynamics of CRS-induced fatigue in mice. Mice could experience increasing fatigue when suffering from increasing intensity of CRS, and could experience severe fatigue when subjected to CRS for 8 h per day for 15 days. As shown in Figure 8, fatigue might be caused by muscle atrophy and mitochondrial dysfunction of the gastrocnemius via the AMPK/PGC-1α signaling pathway. CRS led to mitochondrial damage and blocked protective mitophagy. Our findings provide awareness of the treatment for stress-induced fatigue.

## Data Availability

The data presented in this study are available on request from the corresponding author.
